# Clinical Characteristics and Relevance of Oral *Candida* Biofilm in Tongue Smears

**DOI:** 10.3390/jof7020077

**Published:** 2021-01-22

**Authors:** Eunae Cho, YounJung Park, Ki-Yeol Kim, Dawool Han, Hyun Sil Kim, Jeong-Seung Kwon, Hyung-Joon Ahn

**Affiliations:** 1 Department of Oral Pathology, Oral Cancer Research Institute, Yonsei University College of Dentistry, Seoul 03722, Korea; sandra@yuhs.ac (E.C.); ipodvideo@yuhs.ac (D.H.); KHS@yuhs.ac (H.S.K.); 2 BK21 FOUR Project, Yonsei University College of Dentistry, Seoul 03722, Korea; KKY1004@yuhs.ac; 3 Department of Orofacial Pain and Oral Medicine, Dental Hospital, Yonsei University College of Dentistry, Seoul 03722, Korea; DARKSTAR@yuhs.ac; 4 Department of Dental Education, Yonsei University College of Dentistry, Seoul 03722, Korea

**Keywords:** oral candidiasis, *Candida*, hyphae, biofilm, erythematous candidiasis, atrophic candidiasis, smears, tongue

## Abstract

Dimorphic *Candida* exist as commensal yeast carriages or infiltrate hyphae in the oral cavity. Here, we investigated the clinical relevance of *Candida* hyphae in non-pseudomembranous oral candidiasis (OC) by smears of tongue biofilms. We conducted a retrospective study of 2829 patients who had had tongue smears regardless of OC suspicion. Clinical characteristics were evaluated using a novel method of assessing hyphae. Clinical factors (moderate/severe stimulated pain, pain aggravated by stimulation, tongue dorsum appearance and initial topical antifungal use) were highly significant in the high-grade hyphae group but were statistically similar in the low-grade hyphae and non-observed hyphae group, suggesting low-grade hyphae infection as a subclinical OC state. In addition to erythematous candidiasis (EC), a new subtype named “morphologically normal symptomatic candidiasis” (MNSC) with specific pain patterns and normal tongue morphology was identified. MNSC had a significantly higher proportion of moderate and severe stimulated pain cases than EC. Low unstimulated salivary flow rate (<0.1 mL/min) was found to be a common risk factor in MNSC and EC. In non-pseudomembranous OC, pain patterns were dependent on *Candida* hyphae degree regardless of tongue dorsum morphology. Morphologic differences seen in high-grade hyphae infection were not associated with systemic diseases or nutritional deficiencies.

## 1. Introduction

*Candida* are dimorphic fungus presented as commensal yeast carriages or invasive hyphae [1]. The hyphae phenotype is associated with virulence, epithelial infiltration, tissue damage, keratinization and biofilm formation [2,3,4,5,6,7,8]. Thus, hyphae identification is necessary in detecting oral candidiasis (OC) transition from commensal *Candida* [9]. Recently, the biofilm formation is assumed to have a critical role in OC in addition to hyphae transition [10,11,12]. *Candida* biofilm comprises a sessile-shaped heterogenous ecosystem, which includes *Candida* microorganisms, extracellular matrix and sometimes bacteria [13]. Compared to planktonic *Candida*, biofilm formation induces persistent infection, recurrence and antifungal resistance and is essential for *Candida* pathogenicity in mucosal candidiasis [12,13,14,15].

OC is typically classified according to its clinical manifestations, representatively, pseudomembranous candidiasis (PC) and erythematous candidiasis (EC) [16,17]. In the oral cavity, the tongue is the primary reservoir for *Candida* colonization [18]. *Candida* biofilms are assumed to be causative for PC in the soft tissue, including the tongue [10,19]. However, there is no known association between *Candida* biofilms and non-PC subtypes. Importantly, it is unclear what clinical manifestations, aside from whitish curd-like material (pseudomembrane), are relevant to *Candida* biofilms. In this study, we assessed the tongue biofilm to evaluate the relevance of clinical characteristics and *Candida* hyphae in non-PC subtypes.

## 2. Materials and Methods

### 2.1. Data Collection, Study Design and Variable Definition

This retrospective study, approved by the Institutional Review Board at Yonsei University Dental Hospital (2-2017-0001), was based on medical records and smear slides of patients that had visited Yonsei University Dental Hospital from 2014 to 2019 and had received a tongue smear (*n* = 2829). Clinically confirmed cases of oral PC were later excluded during the study. 

Assessment factors were age, sex, pain characteristics (spontaneous pain [SpP], stimulated pain [StP] and pain difference by stimulation [PDSt]), tongue dorsum morphology, coexisting oral and systemic conditions, pathologic hyphae grade, salivary flow rate, complete blood counts and nutrition blood levels. The criteria of assessment factors are specified in Table 1. Pain characteristics had been evaluated by subjective pain response based on the numerical rating scale (NRS). Ulcerative or erosive oral diseases that could mask tongue pain were excluded during pain record evaluation. Tongue dorsum morphology was assessed by medical records and (if available) previous clinical photos.

Tongue biofilms had been sampled from the dorsal tongue with a wooden tongue depressor and smeared onto glass slides. The slides, which had been fixed with ethyl alcohol and stained with Periodic acid-Schiff (PAS) stain, were put under a light microscope for pathologic examination of *Candida* hyphae.

*Candida* hyphae were evaluated using a novel method established by our group. Hyphae were first divided into the existence of hyphae (H) group and the no observed hyphae (NH) group. The H group was further designated either low-grade hyphae (LGH) or high-grade hyphae (HGH) that was divided by a hyphae value cut-off point selected based on a preliminary study described in Figure S1 and Table S1. We analyzed clinical pain and morphological characteristics with the total *Candida* hyphae value in 48 smeared slides to identify specific correlation patterns. The clinical characteristics did not present a linear correlation pattern with the total hyphae count, but we noticed that certain characteristics, such as StP intensity and PDSt, revealed specific patterns in the range of hyphae values over a cut-off point (Figure S1). Next, we analyzed candidates of the hyphae value cut-off point within the range of 6 to 50, to determine an accurate cut-off point that had the most sensitivity and specificity for clinical relevance—being 10 (Table S1). Based on the cut-off point 10, LGH and HGH were defined as detailed in Table 1. Since total hyphae counts equal and over 10 were uniformly classified as HGH, the total sum of *Candida* hyphae at ten selected high-power fields in the order of highest hyphae aggregations were used for hyphae grade assessment in the final analysis.

Due to the severe imbalance between the NH group and the H (*n* = 135)/HGH (*n* = 69)/LGH (*n* = 66) groups, we randomly selected control cases (*n* = 205) from the NH group within 3 folds of the hyphae group of interest (HGH).

### 2.2. Statistics

The distribution and proportion of the data based on pathologic groups were analyzed by Mann–Whitney U test, Kruskal–Wallis H test and Chi-square test based on the characteristics of the dataset. Hyphae value cut-off points were analyzed in terms of sensitivity, specificity and accuracy. Risk factors were evaluated by simple and multiple logistic regression (odds ratio [OR] and 95% confidence interval [CI]), and the model was evaluated using receiver operating characteristic (ROC) curve analysis. *p* < 0.05 were considered significant. Statistics were analyzed with IBM SPSS Statistics for Windows, Version 25.0. Armonk, NY: IBM Corp.

## 3. Results

### 3.1. Basic Characteristics of the Study

Tongue smears from a total of 2829 patients were primarily included for evaluation. The smears had been routinely conducted in patients with a wide range of chief complaints in the oral soft tissue, regardless of OC suspicion or tongue pain. Chief complaints included but were not limited to oral pain, painless benign or malignant soft tissue lesions, trauma, dry mouth, halitosis or neuropathic disorder in the overall oral cavity.

Among them, *Candida* hyphae were observed in the smears of 244 patients (8.6%). Twenty-three cases yielded insufficient smear material and were excluded. Eight of the 244 patients were confirmed PC and, thus, excluded.

The final dataset (*n* = 340), after exclusion of inappropriate data and random selection, had a median age of 64 years (interquartile range [IQR], 55–73), 79.7% being female (Table 2).

### 3.2. Specific Characteristics of Candida Infection

Table 2 shows the clinical characteristics of the final dataset based on *Candida* hyphae grade. The overall percentages of patients showing moderate and severe StP intensity were significantly higher in the HGH group than in the LGH or NH group (40.6 and 43.5%, 24.2 and 19.7%, 20.5 and 15.1%, respectively, *p* < 0.001). The rate of pain aggravation by stimulation (AggSt) was significantly higher in the HGH group compared to the LGH and NH groups (82.6, 45.5 and 37.1%, respectively, *p* < 0.001). Moreover, a higher proportion of pain alleviation by stimulation (AllSt) was seen in the LGH and NH groups (16.7 and 21.0%, respectively), whereas there was only a single AllSt case (1.4%) in the HGH group. Clinical characteristics based on hyphae grade revealed that moderate/severe StP intensity and AggSt, but not SpP, were significant in the HGH group; these were assumed to be specific characteristics of non-pseudomembranous subtypes of OC. This was supported by the differences in antifungal response between the hyphae groups. Initial topical fluconazole use for 2 weeks was more significantly effective in the HGH group than the LGH group for StP relief (*p* < 0.001), but not SpP relief (*p* = 0.502). Statistically, however, clinical characteristics had a similar distribution in the LGH and NH groups, implying that the hyphae infection in the LHG group reflected a subclinical OC state.

**Figure 1:**
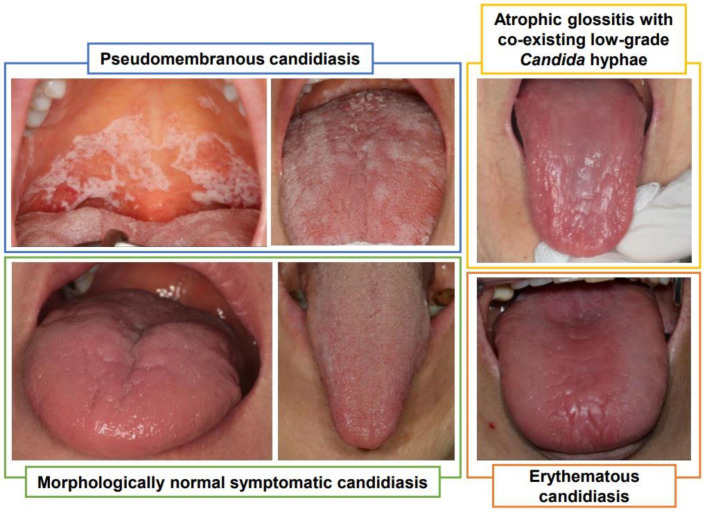
Subtypes of oral candidiasis based on morphologic variants.

Among the tongue dorsal morphologic variants, the atrophic appearance was most specific to the HGH group compared to the LGH and NH groups (47.8, 27.3 and 24.4%, respectively, *p* < 0.01). Despite tongue atrophy being specific to the HGH group, clinically normal tongue morphology predominated in all three hyphae grades, including the HGH group (52.2%). HGH cases with an atrophic tongue morphology were defined as EC, while painful HGH cases with a normal tongue morphology were defined as “morphologically normal symptomatic candidiasis” (MNSC) (Figure 1).

Blue box: pseudomembranous candidiasis with diffuse and easily removable whitish curd-like pseudomembrane in the oral cavity.

Green box: morphologically normal symptomatic candidiasis presenting normal (left) or mild papillary hyperkeratosis (right) on the tongue dorsum with high-grade *Candida* hyphae infection.

Yellow box: Atrophic glossitis with diffuse severe tongue papilla atrophy, low-grade *Candida* hyphae infection and accompanying systemic disorders (low red blood cell count, low hemoglobin level and vitamin B12 deficiency). The mucosal atrophy or moderate intensity of stimulated pain were not relieved by topical anti-fungal agent (fluconazole) use in this patient.

Orange box: erythematous candidiasis presenting partial tongue atrophy with high-grade *Candida* hyphae infection.

### 3.3. Risk Factors of OC Subtypes

The comparison of clinical characteristics in non-PC subtypes is described in Table 3. The proportion of moderate and severe stimulated pain cases were significantly higher in MNSC (97.1%) than EC (72.8%) (*p* < 0.05). The other characteristics were statistically similar between the two groups.

Logistic regression of risk factors in MNSC and EC are detailed in Table 4. Multiple logistic regression models for non-PC subtypes revealed that low unstimulated salivary flow rate, but not stimulated salivary flow rate, was a significant risk factor in both MNSC (OR 5.3, 95% CI 1.8–15.4, *p* < 0.01) and EC (OR 8.2, 95% CI 2.3–28.9, *p* < 0.01) models. Denture was a significant risk factor only in the MNSC model (OR 4.9, 95% CI 1.5–15.6, *p* < 0.01). Other systemic factors, such as complete blood counts and nutrition blood levels, were not risk factors of either MNSC or EC.

## 4. Discussion

The opportunistic nature of *Candida* infection and its various clinical phenotypes poses difficulties during clinical diagnosis and control of OC [16,20,21]. The formation of *Candida* biofilms during infection is assumed to contribute to its diverse clinical characteristics [10,19]. Because clinical and pathologic examinations of OC had been confirmed and widely accepted before the concept of *Candida* biofilms emerged, previous studies on the clinical relevance of *Candida* infection were mainly based on the whole *Candida* carriage or cultured specimens under controlled experimental conditions [18,20,21,22,23,24]. Among existing diagnostic methods for OC, smears are effective in collecting tongue biofilms. *Candida* hyphae, intermixed yeasts, keratinocytes, extracellular matrix and bacteria can be scraped from the mucosal surface onto a pathologic slide for examination [19,25,26,27]. Lack of sensitivity has been frequently mentioned as a major disadvantage of smears, but in our opinion, the insufficient sensitivity is due to a lack of capturing planktonic microorganisms, which are generally commensal *Candida* carriage [15,20]. Hyphae, which indicate active disease progression, attached to and infiltrate the superficial mucosa and require pressured mucosal scraping for appropriate sampling [7,8,21]. Furthermore, biofilms provide their contents with additional surface adherence, which facilitates their collection by smears [28,29,30]. To address the difficulty of evaluating smears quantitatively [20,31], we arranged a novel hyphae assessing method to evaluate the clinical characteristics of non-pseudomembranous OC. High/low hyphae grade discrimination not only revealed specific pain patterns of the disease, but also showed us that the clinical symptoms of *Candida* infection may be non-specific and indistinguishable from other painful or erythematous soft tissue lesions at the low hyphae grade phase.

In addition to the classic PC and EC subtypes, we characterized a previously neglected subtype of OC, provisionally termed MNSC. Although a previous study has noted a few cases of normal tongue appearance in healthy humans with observable *Candida* hyphae [18], this is the first study to suggest that normal tongue morphology in conjunction with pain symptoms is a definite clinical feature in OC. Since OC classification has been primarily clinical-morphology-based, this subtype has gone unheeded in the clinic and may be misdiagnosed as other painful oral diseases (e.g., burning mouth syndrome) with co-existing commensal *Candida* [16,17,32,33]. Contrary to conventional classification criteria, we believe that specific pain symptoms and hyphae grade are the major diagnostic factors for OC, rather than mucosal morphology. Although a previous study reported painful OC without external abnormalities, the evaluation had been based on the whole *Candida* carriage without discrimination of the disease phenotype, hyphae [34].

*Candida*-induced pain has been ambiguously described in the literature, such as burning, sensitive or painful, mostly without clear distinction between SpP and StP [16,20,35]. The association of StP and *Candida* carriage has been noticed in previous studies, but the correlation between StP and active *Candida* hyphae infection or soft tissue *Candida* biofilms has not been examined before [33,34]. Our findings indicated moderate to severe StP as a specific pain characteristic of *Candida* hyphae infection in the tongue biofilm. Moreover, SpP or AllSt, which are known features of burning mouth syndrome or atrophic glossitis caused by nutritional deficiencies, did not show association with high-grade *Candida* hyphae infection [36,37,38,39]. Consistent with our results, other studies have reported antifungal treatment to be ineffective in patients with mainly SpP [33,34]. Altogether, these suggest that concrete discrimination of pain features is informative for clinical differentiation of OC. Interestingly, we observed that MNSC had a more advanced degree of StP intensity than EC. Moreover, there were no painless patients in MNSC, unlike EC. This implied that mucosal atrophy or erythema may not be the major cause of *Candida*-induced pain.

Factors associated with morphologic phenotype diversity are poorly understood in OC. As specific pain symptoms are universal in non-pseudomembranous OC, we further investigated factors associated with clinical morphology in *Candida* infection. Conspicuous hyphae transition and proliferation have been observed in all PC, EC and MNSC, although the degree of direct hyphae/keratinocyte infiltration may be relatively indistinct in EC [21,37,40,41]. *Candida* hyphae are known to induce increased keratin differentiation and pseudomembrane formation [3,21,40], but whether they are responsible for contrasting clinical features of mucosal atrophy or normal differentiation is an intricate issue and rather a paradox. Despite that the presence of tongue atrophy and pain may suggest *Candida* infection, atrophic glossitis can arise from other conditions including salivary hyposecretion, anemia and nutritional deficiencies [17,35,36,37,38,39]. Possible local and systemic risk factors of morphological non-PC subtypes did not differ except for denture use. Although denture use was not statistically significant in EC, it showed a strong tendency as a risk factor as well. These results indicated that co-existing anemia or nutritional deficiencies had minimal impact on atrophic or erythematous morphology in definite OC with high-grade hyphae.

Other than clinical factors, the contents of *Candida* biofilm may contribute to OC morphology phenotypes. Although our retrospective study focused mainly on the clinical relevance of the hyphae content within the smeared tongue material, tongue smears can provide a comprehensive profile of non-hyphae contents in the biofilm. The composition of extracellular matrix, immune cells and bacteria and their interactions with *Candida* organisms within the biofilm may influence the differentiation of underlying epithelial cells [11,27,29,42,43]. Further study of non-*Candida* biofilm contents may lead to systemic insights into the clinical relevance of *Candida* infection.

Various local or systemic conditions have been suggested as causative or predisposing factors of OC [40,41,42,43]. These factors may directly affect fungal microorganisms or may induce an immunosuppressive state or biofilm formation that can enhance *Candida* pathogenesis [42,44,45,46,47,48]. In general, many patients have more than one possible risk factor for OC, and the degree of the risk conditions vary among individuals. Furthermore, the association between *Candida* and possible risk factors can differ based on the method used for fungus measurement, as *Candida* has a dimorphic presence.

*Candida* infection related with denture use is called denture stomatitis. It typically arises on the palate mucosa in contact with the acrylic surface of maxillary dentures [48,49,50,51]. *Candida*-induced denture stomatitis is presented as red, atrophic and sore mucosa at the interface of the denture surface and soft tissue. Co-associated bacteria species have been suggested to aid *Candida* biofilm formation in dentures [52,53,54,55,56,57]. Dentures were significantly involved with MNSC showing normal tongue mucosa in this study. Although erythema and atrophy are characteristic in denture stomatitis, they do not seem to be significant in the tongue mucosa of denture users. The tongue does not have a tight and intimate contact with the denture surface as seen in the palate or gingiva, thus the association between dentures and the tongue *Candida* biofilm is suspected to be indirect. Dentures may contribute as a reservoir for increased oral *Candida* carriage that can lead to *Candida* hyphae and biofilm formation at the tongue in appropriate conditions.

Hyposalivation is one of the most mentioned risk factors of OC. *Candida* carriages have been commonly observed in xerostomia [52,56,57]. Saliva contains antifungal proteins and antibodies that inhibit *Candida* adhesion and colonization and stimulates an innate immune response against *Candida* microorganisms [52,57,58]. We identified that reduced unstimulated saliva, but not stimulated saliva, was a common risk factor in MNSC and EC multiple regression models. Other studies observed that *Candida* count had an inverse relation with both stimulated and unstimulated salivary flow, but the investigations were based on planktonic fungal carriage collected from saliva [24,48,49,58]. Reduced stimulated and unstimulated saliva have different molecule compositions [59], but their pathogenic effect on *Candida* colonization and biofilm formation are not known.

Smoking, alcohol consumption, hypertension, diabetes mellitus and hepatitis B were not risk factors for non-PC subtypes in this study. Chemicals in cigarette smoke has been proposed to induce *Candida* infection and biofilm formation in vitro, but its affects in clinical settings are controversial [18,60,61,62]. *Candida* has been suspected to be related to diabetes in both adults and children [63,64,65,66,67]. In contrast to our results, Bartholomew et al. observed that invasive *Candida* was more frequently identified in diabetic patients than non-diabetic subjects by oral cytologic smears [64]. Sato et al. reported that hypertension and daily alcohol intake were associated with greater carriage of *Candida* albicans in the elderly resided at the post-disaster region, but this was not seen in our results based on invasive *Candida* [68].

## 5. Conclusions

New insights on *Candida* biofilm formation during hyphae transition and infiltration have brought up the need for clinical assessment based on invasive hyphae within the biofilm. In this study, we evaluated the clinical relevance of *Candida* infection in smeared tongue biofilms based on our novel hyphae grade method. We discovered that stimulated pain was a critical and specific characteristic, while tongue mucosal atrophy was a significant but not a specific characteristic in non-PC subtypes. Importantly, we report a morphologically normal, yet symptomatic, OC subtype named “morphologically normal symptomatic candidiasis” as a common non-PC subtype. The morphological differences of MNSC and EC were thought to be minimally affected by clinical risk factors; instead, other factors such as non-fungal components of the *Candida* biofilm may contribute to the diversity of morphologic phenotypes in non-PC subtypes.

## Figures and Tables

**Table 1:** Criteria of clinical and pathologic factors.

Factors	Criteria
Pain characteristics	
Spontaneous pain (SpP)	Intensity: 0–10 (NRS)
Stimulated pain (StP)	Intensity: 0–10 (NRS)
Pain intensity	Mild, <NRS4; moderate, ≤NRS4, <NRS7; severe, 7 ≤ NRS ≤ 10
Pain difference by stimulation (StP-SpP intensity)	Positive value: pain aggravation by stimulation (AggSt)
	0: no pain difference
	Negative value: pain alleviation by stimulation (AllSt)
Tongue dorsum morphology	Atrophy: any partial or full tongue papilla atrophy
	Normal: a pink mucosa with normal tongue papilla or mild keratotic tongue papilla/mucosa
Hyphae grade ^a^	High-grade hyphae (HGH): hyphae value ≥10
	Low-grade hyphae (LGH): hyphae value <10
	No observed hyphae (NH): no hyphae
	Insufficient material: smear slides with fewer than ten high-power fields of valid oral material

Abbreviations: NRS, numeric rating scale. ^a^ Based on the total sum of *Candida* hyphae in ten selected high-power fields in the order of highest hyphae aggregations on smear slides.

**Table 2:** Clinical characteristics based on Candida hyphae grade.

Characteristics	Entire Study	H		NH	*p* Value
					H/NH
		HGH	LGH		HGH/LGH/NH
	Number of subjects/total (%), unless otherwise stated				
Age					
Median (IQR), years	64 (55–73)	68 (60–77)		61 (53–70)	**<0.001**
		70 (61–76)	67 (58–77)		**<0.001**
<49 years	55/340 (16.2)	15/135 (11.1)		40/205 (19.5)	**<0.001**
		7/69 (10.1)	8/66 (12.1)		**<0.001**
50–69 years	174/340 (51.2)	58/135 (43.0)		116/205 (56.6)	
		27/69 (39.1)	31/66 (47.0)		
≤70 years	111/340 (32.6)	62/135 (45.9)		49/205 (23.9)	
		35/69 (50.7)	27/66 (40.9)		
Sex					
Female (%)	271/340 (79.7)	112/135 (83.0)		159/205 (77.6)	0.226
		58/69 (84.1)	54/66 (81.8)		0.455
Pain symptoms					
Presence of SpP	174/340 (51.2)	69/135 (51.1)		105/205 (51.2)	0.984
		38/69 (55.1)	31/66 (47.0)		0.642
SpP intensity, median (IQR), NRS	1 (0–4)	2 (0–4)		1 (0–4)	0.831
		2 (0–5)	0 (0–4)		0.520
Painless	166/340 (48.8)	66/135 (48.9)		100/205 (48.8)	0.584
		31/69 (44.9)	35/66 (53.0)		0.728
Mild ^a^	55/340 (16.2)	23/135 (17.0)		32/205 (15.6)	
		11/69 (15.9)	12/66 (18.2)		
Moderate ^a^	93/340 (27.4)	33/135 (24.4)		60/205 (29.3)	
		20/69 (29.0)	13/66 (19.7)		
Severe ^a^	26/340 (7.6)	13/135 (9.6)		13/205 (6.3)	
		7/69 (10.1)	6/66 (9.1)		
Presence of StP	185/340 (54.4)	98/135 (72.6)		87/205 (42.4)	**<0.001**
		64/69 (92.8)	34/66 (51.5)		**<0.001**
StP intensity, median (IQR), NRS	2.5 (0–6.5)	5 (0–7.5)		0 (0–6)	**<0.001**
		6 (4.5–8)	1.3 (0–6.4)		**<0.001**
Painless	155/340 (45.6)	37/135 (27.4)		118/205 (57.6)	**<0.001**
		5/69 (7.2)	32/66 (48.5)		**<0.001**
Mild ^a^	25/340 (7.4)	11/135 (8.1)		14/205 (6.8)	
		6/69 (8.7)	5/66 (7.6)		
Moderate ^a^	86/340 (25.3)	44/135 (32.6)		42/205 (20.5)	
		28/69 (40.6)	16/66 (24.2)		
Severe ^a^	74/340 (21.8)	43/135 (31.9)		31/205 (15.1)	
		30/69 (43.5)	13/66 (19.7)		
PDSt, median (IQR), NRS	0 (0–2.5)	2 (0–4.5)		0 (0–2)	**<0.001**
		3 (1–5)	0 (0–2.5)		**<0.001**
Presence of AggSt	163/340 (47.9)	87/135 (64.4)		76/205 (37.1)	**<0.001**
		57/69 (82.6)	30/66 (45.5)		**<0.001**
Presence of AllSt	55/340 (16.2)	12/135 (8.9)		43/205 (21.0)	
		1/69 (1.4)	11/66 (16.7)		
No pain difference	122/340 (35.9)	36/135 (26.7)		86/205 (42.0)	
		11/69 (15.9)	25/66 (37.9)		
Tongue dorsal morphology					
Normal	239/340 (70.3)	84/135 (62.2)		155/205 (75.6)	**0.008**
		36/69 (52.2)	48/66 (72.7)		**0.001**
Atrophy	101/340 (29.7)	51/135 (37.8)		50/205 (24.4)	
		33/69 (47.8)	18/66 (27.3)		
Initial antifungal therapy response, topical fluconazole syrup, 2 w					
Effective on SpP ^b^		36/110 (32.7)			
		19/55 (34.5)	17/55 (30.9)		0.824
Non-effective on SpP ^b^		7/110 (6.4)			
		4/55 (7.3)	3/55 (5.5)		
No SpP		67/110 (60.9)			
		32/55 (58.2)	35/55 (63.6)		
Effective on StP ^b^		46/97 (47.4)			
		33/44 (75.0)	13/53 (24.5)		**<0.001**
Non-effective on StP ^b^		16/97 (16.5)			
		8/44 (18.2)	8/53 (15.1)		
No StP		35/97 (36.1)			
		3/44 (6.8)	32/53 (60.4)		

Differences between proportions were determined by the Chi-square test and range distributions were compared by either Mann–Whitney U test or Kruskal–Wallis H test. *p* values < 0.05 are in bold. ^a^ Pain intensity: mild, <NRS4; moderate, ≤NRS4, <NRS7; severe, 7 ≤ NRS ≤ 10. ^b^ Effective: full or partial pain relief. Abbreviations: H, existence of hyphae; NH, no observed hyphae; LGH, low-grade hyphae; HGH, high-grade hyphae; IQR, interquartile range; SpP, spontaneous pain; StP, stimulated pain; AggSt, pain aggravation by stimulation; AllSt, pain alleviation by stimulation; NRS, numeric rating scale.

**Table 3:** Clinical characteristics of morphologically normal symptomatic candidiasis and erythematous candidiasis.

Characteristics	Morphologically Normal Symptomatic OC	Erythematous OC	*p* Value
Number of subjects/total (%), unless otherwise stated			
Age			
<49 years	5/35 (14.3)	2/33 (6.1)	0.432
50–69 years	12/35 (34.3)	15/33 (45.5)	
≤70 years	18/35 (51.4)	16/33 (48.5)	
Sex			
Female (%)	30/35 (85.7)	28/33 (84.8)	0.920
Pain symptoms			
Spontaneous pain intensity			
None	14/35 (40.0)	16/33 (48.5)	0.417
Mild ^a^	4/35 (11.4)	7/33 (21.2)	
Moderate ^a^	13/35 (37.1)	7/33 (21.2)	
Severe ^a^	4/35 (11.4)	3/33 (9.1)	
Stimulated pain intensity			
None	0/35 (0)	4/33 (12.1)	**0.039**
Mild ^a^	1/35 (2.9)	5/33 (15.2)	
Moderate ^a^	16/35 (45.7)	12/33 (36.4)	
Severe ^a^	18/35 (51.4)	12/33 (36.4)	
Presence of AggSt	32/35 (91.4)	25/33 (75.8)	0.182
Presence of AllSt	0/35 (0)	1/33 (3.0)	
No pain difference	3/35 (8.6)	7/33 (21.2)	
Initial antifungal therapy response, topical fluconazole syrup, 2 w			
Effective on SpP ^b^	10/27 (37.0)	9/27 (33.3)	0.958
Non-effective on SpP ^b^	2/27 (7.4)	2/27 (7.4)	
No SpP	15/27 (55.6)	16/27 (59.3)	
Effective on StP ^b^	17/22 (77.3)	16/21 (76.2)	0.285
Non-effective on StP ^b^	5/22 (22.7)	3/21 (14.3)	
No StP	0/22 (0)	2/21 (9.5)	
Accompanied conditions			
Smoking ^c^	1/32 (3.1)	1/32 (3.1)	0.982
Alcohol ^c^	1/32 (3.1)	0/32 (0)	0.313
Denture ^c^	12/32 (37.5)	10/29 (34.5)	0.806
Hypertension	15/35 (42.9)	9/33 (27.3)	0.179
Diabetes mellitus	7/35 (20.0)	4/33 (12.1)	0.378
Hepatitis B	1/35 (2.9)	0/33 (0)	0.328
Salivary flow rate and presence of hyposecretion			
Low USFR ^c^	21/31 (67.7)	16/26 (61.5)	0.625
Low SSFR ^c^	25/31 (80.6)	17/26 (65.4)	0.193
Complete blood counts			
Low RBC ^c^	6/28 (21.4)	7/28 (25.0)	0.752
Low hemoglobin ^c^	5/28 (17.9)	8/28 (28.6)	0.342
Nutrition blood levels			
Vitamin B12 def ^c^	0/25 (0)	1/24 (4.2)	0.302
Folate def ^c^	0/25 (0)	0/24 (0)	…
Zinc def ^c^	1/23 (4.3)	3/24 (12.5)	0.317
Albumin def ^c^	1/26 (3.8)	1/28 (3.6)	0.957
Serum iron def ^c^	3/21 (14.3)	3/18 (16.7)	1.000

Differences between proportions were determined by the chi square test and range distributions were compared by either Mann–Whitney U test or Kruskal–Wallis H test. *p* values <0.05 are in bold. ^a^ Pain intensity: mild, <NRS4; moderate, ≤NRS4, <NRS7; severe, 7 ≤ NRS ≤ 10. ^b^ Effective: full or partial pain relief. ^c^ Standard: denture, full or partial; smoking, daily; alcohol, consumption over 3 times/week; USFR, <0.1 mL/min; SSFR, <0.7 mL/min; RBC, F: <3.8 (10^6^/µL), M: <4.4; (10^6^/µL); hemoglobin, F: <11.7 (g/dL), M: <13.0 (g/dL); vitamin B12, <211 (pg/mL); folate, <3.1 (ng/mL); zinc, <66 (µg/dL); albumin, <3.3 (g/dL); serum iron, <40 (µg/dL). Abbreviations: SpP, spontaneous pain; StP, stimulated pain; AggSt, pain aggravation by stimulation; AllSt, pain alleviation by stimulation; USFR, unstimulated salivary flow rate; SSFR, stimulated salivary flow rate; RBC, red blood cell count; F, female; M, male; def, deficiency; IQR, interquartile range.

**Table 4:** Logistic regression of risk factors in morphologically normal symptomatic candidiasis and erythematous candidiasis.

Variates ^a^	Morphologically Normal Symptomatic OC		Erythematous OC	
	Odds Ratio (95% CI)	*p* Value	Odds Ratio (95% CI)	*p* Value
Simple logistic regression				
Age	2.1 (1.2–3.8)	**0.009**	2.5 (1.4–4.7)	**0.003**
Sex	1.7 (0.6–4.7)	0.281	1.6 (0.6–4.4)	0.347
Smoking	0.4 (0.1–3.4)	0.418	0.4 (0.1–3.3)	0.401
Alcohol	0.5 (0.1–3.7)	0.464	…	
Denture	5.3 (2.3–12.5)	**<0.001**	4.7 (1.9–11.5)	**0.001**
Hypertension	1.9 (0.9–4.0)	0.087	1.0 (0.4–2.2)	0.904
Diabetes mellitus	1.7 (0.7–4.3)	0.250	1.0 (0.3–2.9)	0.928
Hepatitis B	2.0 (0.2–19.6)	0.559	…	
Low USFR	6.6 (2.8–15.4)	**<0.001**	5.0 (2.1–12.1)	**<0.001**
Low SSFR	4.1 (1.6–10.6)	**0.004**	1.9 (0.8–4.5)	0.163
Low RBC	2.1 (0.7–5.6)	0.163	2.5 (1.0–6.6)	0.063
Low hemoglobin	2.6 (0.8–7.8)	0.097	4.7 (1.8–12.6)	**0.002**
Vit B12 def	…		3.6 (0.3–41.1)	0.305
Folate def	…		…	
Zinc def	0.2 (0.03–1.7)	0.150	0.7 (0.2–2.5)	0.582
Albumin def	7.1 (0.4–116.8)	0.171	6.6(0.4–107.9)	0.188
Iron def	8.4 (1.6–44.7)	**0.013**	8.4 (1.6–44.7)	**0.013**
Multiple logistic regression				
Denture	4.9 (1.5–15.6)	**0.007**	3.3 (1.0–10.9)	0.054
Low USFR	5.3 (1.8–15.4)	**0.002**	8.2 (2.3–28.9)	**0.001**
Low SSFR	1.8 (0.5–6.0)	0.342	0.5 (0.1–1.8)	0.297
Low hemoglobin	2.7 (0.6–12.2)	0.205	2.5 (0.6–11.6)	0.231
AUC (IQR)	0.8 (0.7–0.9)	**<0.001**	0.8 (0.7–0.9)	**<0.001**

The NH (no observed hyphae) group is the reference for logistic regression analysis. *p* value <0.05 are in bold. ^a^ Standard: age, <50, 50–69, ≤70, years; sex, female; denture, full or partial; smoking, daily; alcohol, consumption over 3 times/week; USFR, <0.1 mL/min; SSFR, <0.7 mL/min; RBC, F: <3.8 (10^6^/µL), M: <4.4 (10^6^/µL); hemoglobin, F: <11.7 (g/dL), M: <13.0 (g/dL); vitamin B12, <211 (pg/mL); folate, <3.1 (ng/mL); zinc, <66 (µg/dL); albumin, <3.3 (g/dL); serum iron, <40 (µg/dL). Abbreviations: OC, oral candidiasis; CI, confidence interval; F, female; M, male; USFR, unstimulated salivary flow rate; SSFR, stimulated salivary flow rate; def, deficiency; AUC, area under curve; IQR, interquartile range.

## Data Availability

The data presented in this study are available on request from the corresponding author. The data are not publicly available due to the privacy issues.

## Supplementary Materials

The following are available online at https://www.mdpi.com/2309-608X/7/2/77/s1, Figure S1: Preliminary data of clinical characteristic distribution by total hyphae value, Table S1: Sensitivity, specificity and accuracy of hyphae value candidates on preliminary data.
