# Supplementary material for: Clinical Characteristics and Relevance of Oral Candida Biofilm in Tongue Smears
Source: J Fungi (Basel). 2021 Jan 22;7(2):77. doi: 10.3390/jof7020077 (PMC7912297; doi:10.3390/jof7020077)
Supplement: Supplementary file 1 [file jof-07-00077-s001.zip › Supplementary data_sub_rev.pdf]

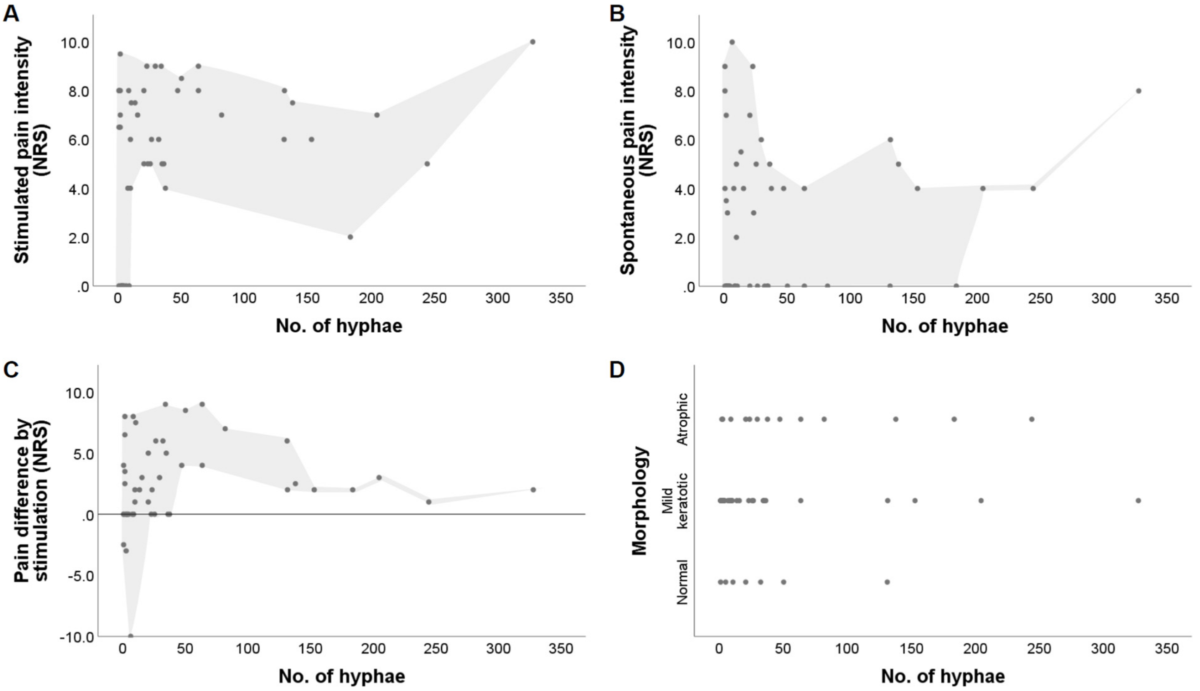

Fig. S1. Preliminary data of clinical characteristic distribution by total hyphae value[a, b]

A. Stimulated pain intensity (NRS): Distributed between NRS4 to 10 in cases with hyphae value over the cut-off point

B. Spontaneous pain intensity (NRS): No specific patterns by hyphae value

C. Pain difference by stimulation (NRS)[c]: Distributed at a positive difference value in cases with hyphae value over the cut-off point

D. Tongue morphologic variants; atrophic and normal variant: No specific patterns by hyphae value

[a]Preliminary data: year 2014-2015, 48 hyphae cases

[b]The gray colored areas present the distribution range of clinical characteristic values.

[c]Pain difference by stimulation was defined as ‘stimulated pain intensity-spontaneous pain intensity (NRS)’.

Hyphae value cut-off point[b]	Presence of stimulated pain			Pain aggravated by stimulation		
	Sensitivity	Specificity	Accuracy	Sensitivity	Specificity	Accuracy
**6**	.821	.777	.813	.844	.563	.750
**8**	.821	.889	.833	.844	.625	.771
**9**	.769	.889	.792	.813	.688	.771
**10**	.769	1	.813	.813	.750	.792
**13**	.692	1	.750	.719	.750	.729
**15**	.667	1	.729	.688	.750	.708
**20**	.641	1	.708	.656	.750	.688
**50**	.692	0	.563	.375	1	.583

Table S1. Sensitivity, specificity and accuracy of hyphae value candidates on preliminary data[a]

[a]Preliminary data : year 2014-2015, 48 hyphae cases

[b]Hyphae grade criteria :

Low-grade : cut-off point > hyphae value, high-grade : cut-off point ≤ hyphae value
