# Supplementary figures and images for: Clinical Characteristics and Relevance of Oral Candida Biofilm in Tongue Smears
Source: J Fungi (Basel). 2021 Jan 22;7(2):77. doi: 10.3390/jof7020077 (PMC7912297; doi:10.3390/jof7020077)

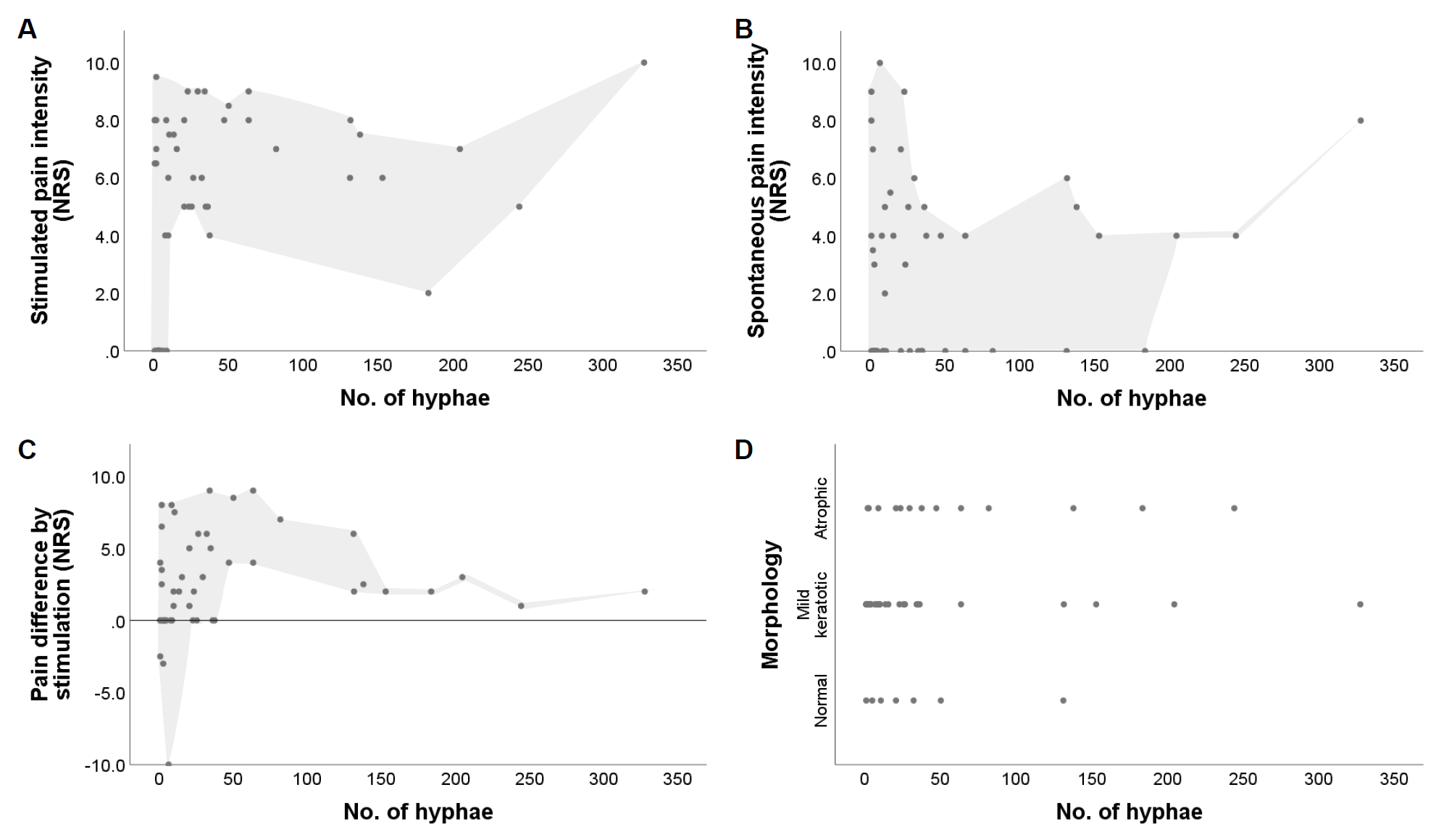

Supplement: Supplementary file 1 [file jof-07-00077-s001.zip › Figure S1.tiff]
